# An Improved SINS Alignment Method Based on Adaptive Cubature Kalman Filter

**DOI:** 10.3390/s19245509

**Published:** 2019-12-13

**Authors:** Yonggang Zhang, Geng Xu, Xin Liu

**Affiliations:** Department of Automation, Harbin Engineering University, Harbin 150001, China; zhangyg@hrbeu.edu.cn (Y.Z.); liushitaocumt@hrbeu.edu.cn (X.L.)

**Keywords:** adaptive Kalman filter, initial alignment, cubature Kalman filter, variational Bayesian method

## Abstract

Initial alignment is critical and indispensable for the inertial navigation system (INS), which determines the initial attitude matrix between the reference navigation frame and the body frame. The conventional initial alignment methods based on the Kalman-like filter require an accurate noise covariance matrix of state and measurement to guarantee the high estimation accuracy. However, in a real-life practical environment, the uncertain noise covariance matrices are often induced by the motion of the carrier and external disturbance. To solve the problem of initial alignment with uncertain noise covariance matrices and a large initial misalignment angle in practical environment, an improved initial alignment method based on an adaptive cubature Kalman filter (ACKF) is proposed in this paper. By virtue of the idea of the variational Bayesian (VB) method, the system state, one step predicted error covariance matrix, and measurement noise covariance matrix of initial alignment are adaptively estimated together. Simulation and vehicle experiment results demonstrate that the proposed method can improve the accuracy of initial alignment compared with existing methods.

## 1. Introduction

The strapdown inertial navigation system (SINS), which is based on a numerical integration procedure, can provide consecutive navigation parameters, including the attitude, velocity, and position for carriers [1,2]. For the dead-reckoning navigation stage, the performance of SINS heavily depends on the accuracy of initial navigation information [3]. The large initial errors (especially the attitude error) will seriously degrade the navigation accuracy. Thus, it is important to estimate the initial attitude of SINS and reduce the initial attitude error. The procedure of determining the initial attitude is named as initial alignment [4,5,6].

Traditional initial alignment methods can be divided into two stages: coarse alignment and fine alignment [7,8]. Coarse alignment, which mainly includes analytic coarse alignment [9], inertial frame coarse alignment [10], and Davenport’s q method based coarse alignment [11,12,13], is to decrease the large misalignment angles into a small range to ensure that the fine alignment has a linear model. However, the average coarse alignment time is hundreds of seconds. When the required time of initial alignment has a stringent restriction, it is difficult to ensure that the misalignment angles have converged to a small range in the coarse alignment stage [4,14]. The condition of a fine alignment with a large misalignment angle may occur. Furthermore, this alignment process needs to switch from coarse alignment to fine alignment at a suitable point of time, which increases the complexity and uncertainty of the alignment process [15]. Therefore, nonlinear filtering algorithm based initial alignment, such as the extended Kalman filter (EKF) [16] and unscented Kalman filter (UKF) [17], has been subsequently developed for decades, to solve the problem of moving state initial alignment with large initial misalignment angles directly. EKF is widely used to solve the nonlinear estimation problem. However, it has low accuracy for a high-dimensional system and a high calculation cost of the Jacobian matrix. UKF was proposed by Julier and Uhlmann in [18]. Based on unscented transformation (UT), UKF can capture the posterior mean and covariance as the second order of the Taylor series of any nonlinearity. Its extensions include marginalized UKF [19] and high order UKF [20]. The cubature Kalman filter (CKF), which is based on the third degree spherical–radial cubature rule, was proposed in [21] and has been used in initial alignment [14]. CKF not only guarantees the accuracy, but also has better stability in a high-dimensional system.

However, the performance of CKF heavily depends on precise prior knowledge of noise. When the carrier is in the motion state, due to the influence of vibration and severe maneuvers, it is difficult to determine the accurate noise covariance value of sensors [22,23,24]. Inaccurate or time varying noise statistical information will degrade the accuracy of initial alignment dramatically. Therefore, there is a great demand to use the adaptive Kalman filter to solve this problem. In [25], an adaptive Kalman filter based on the expectation maximization (EM) method was proposed. However, it met the restriction of needed large windows of data to guarantee reliable estimations. Therefore, it was not suitable in a practical experiment. In [26], the Sage–Husa adaptive Kalman filter based on the maximum a posteriori criterion was used to estimate the statistical properties of noises. Through the introduction of a fading parameter to adjust the filter parameter, it could theoretically estimate the state and measurement noise covariance matrices simultaneously. However, the stability of this filter was critically influenced by the non-positive definiteness of noise covariance matrices, and the capacity of the fading adaptive adjustment factor was limited. Due to the flaws inherent in these adaptive estimation methods, it is urged to adopt the advanced adaptive Kalman filter to meet the requirement. The variational Bayesian (VB) based adaptive Kalman filter was proposed in [27] to further improve the estimation accuracy of unknown noise covariance and has been used in target tracking and cooperative navigation. However, it is not suitable for the initial alignment when the state equation is nonlinear.

Concerning the alignment problem with large misalignment angles and uncertain noise covariance matrices, this paper proposes an adaptive CKF (ACKF) based on the VB method to further improve the accuracy of initial alignment. In this work, the one step predicted covariance matrix and measurement noise covariance matrix are modeled by the inverse Wishart (IW) distribution. The VB method based CKF is then used to approximate the joint posterior probability density function (PDF) of the state, one step predicted error covariance matrix, and measurement noise covariance matrix. The proposed method and existing adaptive filter method are tested based on the vehicle experiment of SINS aided by GPS. Experimental results show that proposed method has better performance than the well known methodologies when the carrier has a severe maneuver.

The outline of this paper is shown as follows. The mathematical model of initial alignment is given in Section 2. In Section 3, the ACKF algorithm is designed for the application of initial alignment. The simulation and vehicle experiment are conducted to verify the effectiveness of the proposed ACKF in Section 4. The conclusions are drawn in Section 5.

## 2. Nonlinear Model of Initial Alignment

**Figure 1:**
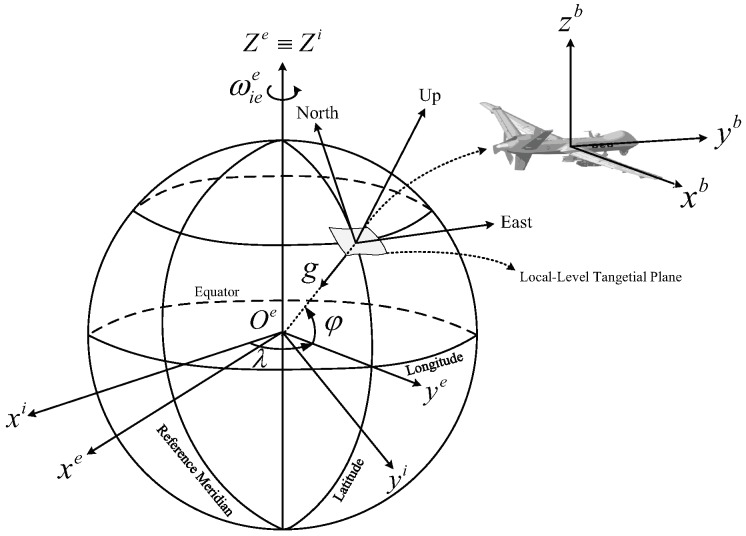
The schematic diagram of the frames.

Firstly, the definitions of the frame are given in this section. The inertial non-rotating frame is denoted by *i*. The Earth-fixed frame is denoted by *e*. The geographic frame is denoted by *t*. The body frame of SINS is denoted as *b*, which is the right-forward-up (R-F-U) orientation. *n* and $n^{\prime }$ represent the navigation frame and the calculated navigation frame, respectively, which are the east-north-up (E-N-U) orientation. The schematic diagram of these frames is shown in Figure 1.

The standard state equation of initial alignment is the same as the error model of SINS [4]. Considering the application of carborne or shipborne equipment, the height error of the carrier is neglected. The nonlinear error equation for the moving state of SINS is given as follows:

(1) $$\left\{\begin{array}{c} \dot{\phi }=C_{\phi }^{-1}\left[\left(I-C_{n}^{n^{\prime }}\right){\tilde{\omega }}_{in}^{n}+C_{n}^{n^{\prime }}\delta \omega_{in}^{n}-C_{b}^{n^{\prime }}\left(\varepsilon^{b}+\eta_{g}^{b}\right)\right]\\ \delta {\dot{v}}^{n}=\left[I-{\left(C_{n}^{n^{\prime }}\right)}^{T}\right]C_{b}^{n^{\prime }}{\tilde{f}}_{ib}^{b}-\left(2{\tilde{\omega }}_{ie}^{n}+{\tilde{\omega }}_{en}^{n}\right)\times \delta v^{n}\\ \;\;-\left(2\delta \omega_{ie}^{n}+\delta \omega_{en}^{n}\right)\times \left({\tilde{v}}^{n}-\delta v^{n}\right)+{\left(C_{n}^{n^{\prime }}\right)}^{T}C_{b}^{n^{\prime }}\left(\nabla^{b}+\eta_{a}^{b}\right)\\ \delta \dot{L}=\frac{\delta v_{N}^{n}}{R_{e}}\\ \delta \dot{\varphi }=\frac{\sec\tilde{L}}{R_{e}}\delta v_{E}^{n}+\frac{{\tilde{v}}_{E}^{n}\sec\tilde{L}\tan\tilde{L}}{R_{e}}\delta L\\ {\dot{\varepsilon }}^{b}=0,{\dot{\nabla }}^{b}=0 \end{array}\right.$$

with:

(2) $$C_{\phi }^{-1}=\frac{1}{\cos\phi_{x}}\left[\begin{array}{ccc} \cos\phi_{x}\cos\phi_{y} & 0 & \cos\phi_{x}\sin\phi_{y}\\ \sin\phi_{x}\sin\phi_{y} & \cos\phi_{x} & -\sin\phi_{x}\cos\phi_{y}\\ -\sin\phi_{y} & 0 & \cos\phi_{y} \end{array}\right]$$

where the misalignment angles $\phi =[\phi_{x};\phi_{y};\phi_{z}]$ are the attitude error angle between frame *n* and frame $n^{\prime }$. $\delta v^{n}=\left[\delta v_{E}^{n};\delta v_{N}^{n}\right]$ is the error of calculated velocity ${\tilde{v}}^{n}$. δL and δφ are the errors of calculated latitude $\tilde{L}$ and longitude $\tilde{\varphi }$. $\varepsilon^{b}$ is the gyro constant drift. $\eta_{g}^{b}$ is the gyroscope random noise. $\nabla^{b}$ is the accelerometer bias. $\eta_{a}^{b}$ is the accelerometer random noise. $R_{e}$ is the radius of the Earth. (·)× denotes the 3×3 skew symmetric matrix. ${\left[\cdot \right]}^{T}$ denotes the transposition of the matrix. The attitude error matrix $C_{n}^{n^{\prime }}$ is formulated as follows:

(3) $$C_{n}^{n^{\prime }}=\left[\begin{array}{ccc} \cos\phi_{y} & 0 & -\sin\phi_{y}\\ 0 & 1 & 0\\ \sin\phi_{y} & 0 & \cos\phi_{y} \end{array}\right]\left[\begin{array}{ccc} 1 & 0 & 0\\ 0 & \cos\phi_{x} & \sin\phi_{x}\\ 0 & -\sin\phi_{x} & \cos\phi_{x} \end{array}\right]\left[\begin{array}{ccc} \cos\phi_{z} & \sin\phi_{z} & 0\\ -\sin\phi_{z} & \cos\phi_{z} & 0\\ 0 & 0 & 1 \end{array}\right]$$

The erroneous ${\tilde{\omega }}_{ie}^{n}$ and ${\tilde{\omega }}_{en}^{n}$ are given as follows:

(4) $${\tilde{\omega }}_{ie}^{n}=\left[\begin{array}{c} 0;\omega_{ie}\cos\tilde{L};\omega_{ie}\sin\tilde{L} \end{array}\right]$$

(5) $${\tilde{\omega }}_{en}^{n}=\left[\begin{array}{c} -\frac{{\tilde{v}}_{N}^{n}}{R_{e}};\frac{{\tilde{v}}_{E}^{n}}{R_{e}};\frac{{\tilde{v}}_{E}^{n}}{R_{e}}\tan\tilde{L} \end{array}\right]$$

where $\omega_{ie}$ is the Earth’s rotation angular velocity. The errors of ${\tilde{\omega }}_{ie}^{n}$ and ${\tilde{\omega }}_{en}^{n}$ are given as follows:

(6) $$\delta \omega_{ie}^{n}=\left[\begin{array}{c} 0;-\omega_{ie}\sin\tilde{L}\delta L;\omega_{ie}\cos\tilde{L}\delta L \end{array}\right]$$

(7) $$\delta \omega_{en}^{n}=\left[\begin{array}{c} -\frac{\delta v_{N}^{n}}{R_{e}};\frac{\delta v_{E}^{n}}{R_{e}};\frac{\delta v_{E}^{n}\tan L+{\tilde{v}}_{E}^{n}\sec^{2}L\delta L}{R_{e}} \end{array}\right]$$

Therefore, ${\tilde{\omega }}_{in}^{n}$ and $\delta \omega_{in}^{n}$ are given as follows:

(8) $${\tilde{\omega }}_{in}^{n}={\tilde{\omega }}_{ie}^{n}+{\tilde{\omega }}_{en}^{n}$$

(9) $$\delta \omega_{in}^{n}=\delta \omega_{ie}^{n}+\delta \omega_{en}^{n}$$

The velocity and position differences between SINS and aiding equipment such as GPS are selected as the measurement, which is formulated as:

(10) $$\left\{\begin{array}{c} z_{v}=\left[\begin{array}{c} \delta v_{E}^{n};\delta v_{N}^{n} \end{array}\right]+\nu_{v}\\ z_{P}=\left[\begin{array}{c} \delta L;\delta \lambda \end{array}\right]+\nu_{P} \end{array}\right.$$

where $\nu_{v}$ and $\nu_{P}$ are velocity measurement noise and position measurement noise, respectively.

The state vector is defined as $x=[\phi_{x};\phi_{y};\phi_{z};\delta v_{E}^{n};\delta v_{N}^{n};$$\delta L;\delta \lambda ;\varepsilon_{x}^{s};\varepsilon_{y}^{s};\varepsilon_{z}^{s};\nabla_{x}^{s};\nabla_{y}^{s}]$. The state noise vector is defined as $w=[\eta_{g}^{s};\eta_{a}^{s};0_{7\times 1}]$. The measurement vector and noise vector are defined as $z=[z_{v};z_{P}]$ and $\nu =[\nu_{v};\nu_{P}]$, respectively. By discretizing the continuous equations, the standard discrete state error equation and measurement equation are formulated as:

(11) $$\left\{\begin{array}{c} x_{k}=f\left(x_{k-1}\right)+g\left(x_{k-1}\right)w_{k-1}\\ z_{k}=h\left(x_{k}\right)+\nu_{k} \end{array}\right.$$

where f(·) and g(·) are the nonlinear functions, which are formulated based on (1), and the measurement model is a linear function,

(12) $$h\left(x_{k}\right)=Hx_{k}=\left[0_{4\times 3},I_{4\times 4},0_{4\times 5}\right]x_{k}$$

$w_{k}$ and $\nu_{k}$ are uncorrelated Gaussian white noises with mean value 0 and covariance $Q_{k}$ and $R_{k}$, respectively. For the sake of simplification in the next sections, we assume that the dimensions of $x_{k}$ and $w_{k}$ are $n_{x}$ and the dimensions of $z_{k}$ and $\nu_{k}$ are $n_{z}$.

The standard nonlinear initial alignment model can be obtained by Equation (11). It can be seen that the initial alignment state model has high dimensions and that the states are coupling strongly. Generally, the state noise and measurement noise of initial alignment are set as Gaussian white noise. However, the severe maneuver and the external disturbance will induce the uncertain noise covariance matrices of SINS, which will degrade the accuracy of initial alignment. In the next section, the adaptive CKF is proposed to solve the problem mentioned above.

## 3. Adaptive Cubature Kalman Filter

In this section, a novel adaptive CKF is proposed to solve the estimation problem with the uncertain noise covariance matrix. Without loss of generality, we introduce this method based on the standard nonlinear model with the nonlinear state and measurement functions.

### 3.1. Gaussian Kalman Filter and Cubature Kalman Filter

The Gaussian filter is the main method to solve the nonlinear estimation, which has two key assumptions, that the one step predicted PDFs of the state and measurement are Gaussian, i.e.,

(13) $$p\left(x_{k}|z_{1:k-1}\right)=N\left(x_{k};{\hat{x}}_{k|k-1},P_{k|k-1}\right)$$

where ${\hat{x}}_{k|k-1}$ and $P_{k|k-1}$ denote the mean and variance of $p\left(x_{k}|z_{1:k-1}\right)$.

(14) $$p\left(z_{k}|z_{1:k-1}\right)=N\left(z_{k};{\hat{z}}_{k|k-1},P_{k|k-1}^{zz}\right)$$

where ${\hat{z}}_{k|k-1}$ and $P_{k|k-1}^{zz}$ denote the mean and variance of $p\left(z_{k}|z_{1:k-1}\right)$.

Obviously, the joint one step predicted PDF of the state and measurement $p\left(x_{k},z_{k}|z_{1:k-1}\right)$ is also Gaussian, i.e.,

(15) $$p\left(x_{k},z_{k}|z_{1:k-1}\right)=N\left(\left[\begin{array}{c} x_{k}\\ z_{k} \end{array}\right];\left[\begin{array}{c} {\hat{x}}_{k|k-1}\\ {\hat{z}}_{k|k-1} \end{array}\right],\left[\begin{array}{cc} P_{k|k-1} & P_{k|k-1}^{xz}\\ {\left(P_{k|k-1}^{xz}\right)}^{T} & P_{k|k-1}^{zz} \end{array}\right]\right)$$

where $P_{k|k-1}^{xz}$ is the covariance of $x_{k}$ and $z_{k}$. Based on (14) and (15), in the Bayesian theorem, the posterior PDF of $x_{k}$ is also Gaussian, i.e.,

(16) $$p\left(x_{k}|z_{1:k}\right)=\frac{p\left(x_{k},z_{k}|z_{1:k-1}\right)}{p\left(z_{k}|z_{1:k-1}\right)}=N\left(x_{k};{\hat{x}}_{k|k},P_{k|k}\right)$$

where ${\hat{x}}_{k|k}$ and $P_{k|k}$ denote the mean and variance of $p\left(x_{k}|z_{1:k}\right)$. ${\hat{x}}_{k|k}$ and $P_{k|k}$ are derived as follows:

(17) $$K_{k}=P_{k|k-1}^{zz}{\left(P_{k|k-1}^{xz}\right)}^{-1}$$

(18) $${\hat{x}}_{k|k}={\hat{x}}_{k|k-1}+K_{k}\left(z_{k}-{\hat{z}}_{k|k-1}\right)$$

(19) $$P_{k|k}=P_{k|k-1}-K_{k}P_{k|k-1}^{zz}K_{k}^{T}$$

where $K_{k}$ is the filter gain, and the other parameters are calculated as follows:

(20) $${\hat{x}}_{k|k-1}=E\left[f\left(x_{k-1}\right)|z_{1:k-1}\right]=\int f\left(x_{k-1}\right)N\left(x_{k-1};{\hat{x}}_{k-1|k-1},P_{k-1|k-1}\right)dx_{k-1}$$

(21) $$\begin{array}{cc} P_{k|k-1}= & E\left[\left(x_{k}-{\hat{x}}_{k|k-1}\right){\left(x_{k}-{\hat{x}}_{k|k-1}\right)}^{T}|z_{1:k-1}\right]\\ = & \int \left(f\left(x_{k-1}\right)-{\hat{x}}_{k|k-1}\right){\left(f\left(x_{k-1}\right)-{\hat{x}}_{k|k-1}\right)}^{T}N\left(x_{k-1};{\hat{x}}_{k-1|k-1},P_{k-1|k-1}\right)dx_{k-1}\\ \; & +g\left({\hat{x}}_{k|k-1}\right)Q_{k-1}g{\left({\hat{x}}_{k|k-1}\right)}^{T} \end{array}$$

(22) $${\hat{z}}_{k|k-1}=E\left[h\left(x_{k}\right)|z_{1:k-1}\right]=\int h\left(x_{k}\right)N\left(x_{k};{\hat{x}}_{k|k-1},P_{k|k-1}\right)dx_{k}$$

(23) $$\begin{array}{cc} P_{k|k-1}^{zz}= & E\left[\left(z_{k}-{\hat{z}}_{k|k-1}\right){\left(z_{k}-{\hat{z}}_{k|k-1}\right)}^{T}|z_{1:k-1}\right]\\ = & \int \left(h\left(x_{k}\right)-{\hat{z}}_{k|k-1}\right){\left(h\left(x_{k}\right)-{\hat{z}}_{k|k-1}\right)}^{T}N\left(x_{k};{\hat{x}}_{k|k-1},P_{k|k-1}\right)dx_{k}+R_{k} \end{array}$$

(24) $$\begin{array}{cc} P_{k|k-1}^{xz}= & E\left[\left(x_{k}-{\hat{x}}_{k|k-1}\right){\left(z_{k}-{\hat{z}}_{k|k-1}\right)}^{T}|z_{1:k-1}\right]\\ = & \int \left(x_{k}-{\hat{x}}_{k|k-1}\right){\left(h\left(x_{k}\right)-{\hat{z}}_{k|k-1}\right)}^{T}N\left(x_{k};{\hat{x}}_{k|k-1},P_{k|k-1}\right)dx_{k} \end{array}$$

where E[·] means the expectation operation.

From (20) to (24), the general framework of the Gaussian filter is established, and the core idea of the Gaussian filter is to calculate Gaussian weighted integrals. Due to the nonlinearity of f(·) and h(·), it is difficult to obtain the accurate numerical solution of (20)–(24), and the approximation solution is necessary, i.e.,

(25) $$\int f\left(x\right)N\left(x;a,b\right)dx\approx \sum_{j=1}^{N}W_{j}f\left(x_{j}\right)$$

where $x_{j}$ and $W_{j}$ are the sampling points and corresponding weights of x.

CKF, which is a typical Gaussian filter, uses the third degree spherical–radial cubature rule to obtain these weighted samples. In (20), the cubature points of $x_{k-1}$ are selected based on ${\hat{x}}_{k-1|k-1}$ and $P_{k-1|k-1}$. These cubature points are defined as follows:

(26) $$\left\{\begin{array}{c} \chi_{k-1}^{\left(j\right)}={\hat{x}}_{k-1|k-1}+\sqrt{n_{x}}{\left(\sqrt{P_{k-1|k-1}}\right)}_{\left(j\right)},\;\left(j=1,2,\cdots ,n_{x}\right)\\ \chi_{k-1}^{\left(j\right)}={\hat{x}}_{k-1|k-1}-\sqrt{n_{x}}{\left(\sqrt{P_{k-1|k-1}}\right)}_{\left(j\right)},\;\left(j=n_{x}+1,n_{x}+2,\cdots ,2n_{x}\right) \end{array}\right.$$

where ${\left(A\right)}_{\left(j\right)}$ denotes the *j*th column of *A*. Propagating the cubature points of $x_{k-1}$ by f(·), the state one step predicted mean ${\hat{x}}_{k|k-1}$ and covariance $P_{k|k-1}$ can be obtained as follows based on (20) and (21):

(27) $$\chi_{k|k-1}^{x(j)}=f\left(\chi_{k-1}^{\left(j\right)}\right),\left(j=1,2,\cdots ,2n_{x}\right)$$

(28) $${\hat{x}}_{k|k-1}=\frac{1}{2n_{x}}\sum_{j=1}^{2n_{x}}\chi_{k|k-1}^{x(j)}$$

(29) $$P_{k|k-1}=\frac{1}{2n_{x}}\sum_{j=1}^{2n_{x}}\left[\left(\chi_{k|k-1}^{x(j)}-{\hat{x}}_{k|k-1}\right){\left(\chi_{k|k-1}^{x(j)}-{\hat{x}}_{k|k-1}\right)}^{T}\right]+g\left({\hat{x}}_{k|k-1}\right)Q_{k-1}g{\left({\hat{x}}_{k|k-1}\right)}^{T}$$

Furthermore, the cubature points of $x_{k}$ based on ${\hat{x}}_{k|k-1}$ and $P_{k|k-1}$ are selected as follows:

(30) $$\left\{\begin{array}{c} \chi_{k|k-1}^{\left(j\right)}={\hat{x}}_{k|k-1}+\sqrt{n_{x}}{\left(\sqrt{P_{k|k-1}}\right)}_{\left(j\right)},\;\left(j=1,2,\cdots ,n_{x}\right)\\ \chi_{k|k-1}^{\left(j\right)}={\hat{x}}_{k|k-1}-\sqrt{n_{x}}{\left(\sqrt{P_{k|k-1}}\right)}_{\left(j\right)},\;\left(j=n_{x}+1,n_{x}+2,\cdots ,2n_{x}\right) \end{array}\right.$$

Propagating the cubature points of $x_{k}$ by h(·), the measurement one step predicted mean and covariance can be obtained as follows:

(31) $$Z_{k|k-1}^{\left(j\right)}=h\left(\chi_{k|k-1}^{\left(j\right)}\right),\left(j=1,2,\cdots ,2n_{x}\right)$$

(32) $${\hat{z}}_{k|k-1}=\frac{1}{2n_{x}}\sum_{j=1}^{2n_{x}}Z_{k|k-1}^{\left(j\right)}$$

(33) $$P_{k|k-1}^{zz}=\frac{1}{2n_{x}}\sum_{j=1}^{2n_{x}}\left[\left(Z_{k|k-1}^{\left(j\right)}-{\hat{z}}_{k|k-1}\right){\left(Z_{k|k-1}^{\left(j\right)}-{\hat{z}}_{k|k-1}\right)}^{T}\right]+R_{k}$$

(34) $$P_{k|k-1}^{xz}=\frac{1}{2n_{x}}\sum_{j=1}^{2n_{x}}\left[\left(\chi_{k|k-1}^{\left(j\right)}-{\hat{x}}_{k|k-1}\right){\left(Z_{k|k-1}^{\left(j\right)}-{\hat{z}}_{k|k-1}\right)}^{T}\right]$$

Filter gain $K_{k}$ and measurement update are given as follows:

(35) $$K_{k}=P_{k|k-1}^{xz}{\left(P_{k|k-1}^{zz}\right)}^{-1}$$

(36) $${\hat{x}}_{k|k}={\hat{x}}_{k|k-1}+K_{k}\left(z_{k}-{\hat{z}}_{k|k-1}\right)$$

(37) $$P_{k|k}=P_{k|k-1}-K_{k}P_{k|k-1}^{zz}K_{k}^{T}$$

### 3.2. The Proposed Adaptive Cubature Kalman Filter

When the state noise covariance $Q_{k}$ and measurement noise covariance $R_{k}$ are unknown or inaccurate, the estimation accuracy of CKF may degrade or diverge. Because the one step predicted state error covariance $P_{k|k-1}$ is influenced by the inaccurate $Q_{k}$, it is easier to estimate $P_{k|k-1}$ than $Q_{k}$. Therefore, in our works, the state, one step predicted state error covariance $P_{k|k-1}$ and $R_{k}$ are jointly estimated to improve the accuracy of CKF with inaccurate noise statistical properties.

In the frame of Bayesian probability theory, the conjugate prior distribution is selected to guarantee the unified form of the prior and posterior distribution. For the Gaussian distribution with known mean, the standard inverse Wishart (IW) PDF is always used as the conjugate prior distribution. The IW PDF is formulated as follows:

(38) $$\mathrm{IW}\left(B;\zeta ,\Psi \right)=\frac{{\left|\Psi \right|}^{\zeta /2}{\left|B\right|}^{-(\zeta +d+1)/2}e^{-\mathrm{tr}\left(\Psi B^{-1}\right)/2}}{2^{d\zeta /2}\Gamma_{d}\left(\zeta /2\right)}$$

where B is positive definite random matrix, Ψ is the inverse scale matrix, ζ is the degrees of freedom (dof) parameter, *d* is the dimension of B, tr(·) is the trace calculation, and $\Gamma_{d}\left(\cdot \right)$ is the *d*-variate Gamma function. When ζ>d+1, the mean of B is shown as follows:

(39) $$E\left[B\right]=\frac{\Psi }{{\left(\zeta -d-1\right)}^{-1}}$$

Therefore, the prior distribution $p(P_{k|k-1}|z_{1:k-1})$ and $p(R_{k}|z_{1:k-1})$ are modeled as follows:

(40) $$p\left(P_{k|k-1}|z_{1:k-1}\right)=\mathrm{IW}\left(P_{k|k-1};{\hat{t}}_{k|k-1},{\hat{T}}_{k|k-1}\right)$$

(41) $$p\left(R_{k}|z_{1:k-1}\right)=\mathrm{IW}\left(R_{k};{\hat{u}}_{k|k-1},{\hat{U}}_{k|k-1}\right)$$

where ${\hat{t}}_{k|k-1}$ and ${\hat{u}}_{k|k-1}$ are dof parameters and ${\hat{T}}_{k|k-1}$ and ${\hat{U}}_{k|k-1}$ are inverse scale matrices.

The mean value of $P_{k|k-1}$ is set as nominal ${\tilde{P}}_{k|k-1}$, determined by:

(42) $${\tilde{P}}_{k|k-1}=\frac{1}{2n_{x}}\sum_{j=1}^{2n_{x}}\left[\left(\chi_{k|k-1}^{x(j)}-{\hat{x}}_{k|k-1}\right){\left(\chi_{k|k-1}^{x(j)}-{\hat{x}}_{k|k-1}\right)}^{T}\right]+g\left({\hat{x}}_{k|k-1}\right){\tilde{Q}}_{k-1}g{\left({\hat{x}}_{k|k-1}\right)}^{T}$$

where ${\tilde{Q}}_{k-1}$ is the nominal state noise covariance matrix, which means an inaccurate value.

Let:

(43) $$\frac{{\hat{T}}_{k|k-1}}{{\hat{t}}_{k|k-1}-n_{x}-1}={\tilde{P}}_{k|k-1}$$

and set ${\hat{t}}_{k|k-1}=n_{x}+\tau +1$, where τ is a tuning parameter. We can obtain:

(44) $${\hat{T}}_{k|k-1}=\tau {\tilde{P}}_{k|k-1}$$

According to the Bayesian theorem, $p\left(R_{k}|z_{1:k-1}\right)$ is formulated as:

(45) $$p\left(R_{k}|z_{1:k-1}\right)=\int p\left(R_{k}|R_{k-1}\right)p\left(R_{k-1}|z_{1:k-1}\right)dR_{k-1}$$

where $p\left(R_{k-1}|z_{1:k-1}\right)$ is the posterior PDF of $R_{k-1}$. Because the posterior and prior PDF of $R_{k-1}$ has the same distribution, the posterior PDF of $R_{k-1}$ is also formulated as the inverse Wishart distribution, as follows:

(46) $$p\left(R_{k-1}|z_{1:k-1}\right)=\mathrm{IW}\left(R_{k-1};{\hat{u}}_{k|k-1},{\hat{U}}_{k|k-1}\right)$$

Because of the unknown dynamic model of $p\left(R_{k}|R_{k-1}\right)$, we selected a forgetting factor ξ∈(0,1] to spread the previous posterior to the current prior, and the prior parameters in (41) are written as follows:

(47) $${\hat{u}}_{k|k-1}=\xi \left({\hat{u}}_{k-1|k-1}-n_{z}-1\right)+n_{z}+1$$

(48) $${\hat{U}}_{k|k-1}=\xi {\hat{U}}_{k-1|k-1}$$

The initial $R_{0}$ is also assumed as an inverse Wishart PDF, i.e., $p\left(R_{0}\right)=\mathrm{IW}\left(R_{0};{\hat{u}}_{0|0},{\hat{U}}_{0|0}\right)$, where the mean value of $R_{0}$ is set as the initial nominal ${\tilde{R}}_{0}$:

(49) $$\frac{{\hat{U}}_{0|0}}{{\hat{u}}_{0|0}-n_{z}-1}={\tilde{R}}_{0}$$

In order to estimate the state $x_{k}$, one step predicted state error covariance $P_{k|k-1}$ and $R_{k}$, their joint posterior PDF $p\left(x_{k},P_{k|k-1},R_{k}|z_{1:k}\right)$ is calculated. Due to the coupling of these parameters, the analytical solution cannot be obtained. Therefore, the VB method is used to solve the estimation problem in coupling.

(50) $$p\left(x_{k},P_{k|k-1},R_{k}|z_{1:k}\right)\approx q\left(x_{k}\right)q\left(P_{k|k-1}\right)q\left(R_{k}\right)$$

$\left\{q\left(x_{k}\right),q\left(P_{k|k-1}\right),q\left(R_{k}\right)\right\}$ are calculated by minimizing the Kullback–Leibler divergence (KLD):

(51) $$\left\{q\left(x_{k}\right),q\left(P_{k|k-1}\right),q\left(R_{k}\right)\right\}=\arg\min KLD\left(q\left(x_{k}\right),q\left(P_{k|k-1}\right),q\left(R_{k}\right)∥p\left(x_{k},P_{k|k-1},R_{k}|z_{1:k}\right)\right)$$

The optimal solution of (51) is given by:

(52) $$\log q\left(\alpha \right)=E_{\Xi^{\left(-\alpha \right)}}\left[\log p\left(\Xi ,z_{1:k}\right)\right]+c_{\alpha }$$

(53) $$\Xi ≜\left\{x_{k},P_{k|k-1},R_{k}\right\}$$

where log(·) means the logarithmic function, α is the arbitrary element of Ξ, $\Xi^{\left(-\alpha \right)}$ contains all elements in Ξ except for α, and $c_{\alpha }$ means the constant dependent on α. According to the Bayesian theorem, the joint PDF $p(\Xi ,z_{1:k})$ is factored as:

(54) $$p\left(\Xi ,z_{1:k}\right)=p\left(z_{k}|x_{k},R_{k}\right)p\left(x_{k}|z_{1:k-1},P_{k|k-1}\right)p\left(P_{k|k-1}|z_{1:k-1}\right)p\left(R_{k}|z_{1:k-1}\right)p\left(z_{1:k-1}\right)$$

where likelihood PDF $p\left(z_{k}|x_{k}\right)$ is assumed as a normal distribution.

(55) $$p\left(z_{k}|x_{k}\right)=N\left(z_{k};h\left(x_{k}\right),R_{k}\right)$$

Substituting (13), (38), (41), and (55) into (54), we have:

(56) $$\begin{array}{cc} p(\Xi ,z_{1:k})= & N\left(z_{k};h\left(x_{k}\right),R_{k}\right)N\left(x_{k};{\hat{x}}_{k|k-1},P_{k|k-1}\right)\mathrm{IW}\left(P_{k|k-1};{\hat{t}}_{k|k-1},{\hat{T}}_{k|k-1}\right)\\ \; & \times \mathrm{IW}\left(R_{k};{\hat{u}}_{k|k-1},{\hat{U}}_{k|k-1}\right)p\left(z_{1:k-1}\right) \end{array}$$

Taking the logarithm on both sides of (56), the normal distribution N(A;a,Σ) and IW distribution IW(B;ζ,Ψ) are formulated as follows:

(57) $$\begin{array}{cc} \log(N(A;a,\Sigma )) & =\log\left\{\frac{1}{\sqrt{2\pi }{\left|\Sigma \right|}^{\frac{1}{2}}}e^{-\frac{1}{2}{\left(A-a\right)}^{T}\Sigma^{-1}\left(A-a\right)}\right\}\\ \; & =-\frac{1}{2}\log\left|\Sigma \right|-\frac{1}{2}{\left(A-a\right)}^{T}\Sigma^{-1}\left(A-a\right)+\log\frac{1}{\sqrt{2\pi }}\\ \; & =-\frac{1}{2}\log\left|\Sigma \right|-\frac{1}{2}{\left(A-a\right)}^{T}\Sigma^{-1}\left(A-a\right)+c_{A} \end{array}$$

(58) $$\begin{array}{cc} \log(\mathrm{IW}(B;\zeta ,\Psi ))= & \log\left\{\frac{{\left|\Psi \right|}^{\zeta /2}{\left|B\right|}^{-(\zeta +d+1)/2}e^{-\mathrm{tr}\left(\Psi B^{-1}\right)/2}}{2^{d\zeta /2}\Gamma_{d}\left(\zeta /2\right)}\right\}\\ = & -\frac{\left(\zeta +d+1\right)}{2}\log|B|-\frac{1}{2}\mathrm{tr}\left(\Psi B^{-1}\right)+\frac{\zeta }{2}\log\left|\Psi \right|\\ \; & -\frac{d\zeta }{2}\log2-\log\Gamma_{d}\left(\zeta /2\right)\\ = & -\frac{\left(\zeta +d+1\right)}{2}\log\left|B\right|-\frac{1}{2}\mathrm{tr}\left(\Psi B^{-1}\right)+c_{B} \end{array}$$

According to (57) and (58), $\log(p\left(\Xi ,z_{1:k}\right))$ is formulated as:

(59) $$\begin{array}{cc} \log(p\left(\Xi ,z_{1:k}\right))= & -\frac{1}{2}{\left(z_{k}-h\left(x_{k}\right)\right)}^{T}R_{k}^{-1}\left(z_{k}-h\left(x_{k}\right)\right)\\ \; & -\frac{1}{2}\left({\hat{u}}_{k|k-1}+n_{z}+2\right)\log\left|R_{k}\right|-\frac{1}{2}\mathrm{tr}\left(U_{k|k-1}R_{k}^{-1}\right)\\ \; & -\frac{1}{2}\left({\hat{t}}_{k|k-1}+n_{x}+2\right)\log\left|P_{k|k-1}\right|-\frac{1}{2}\mathrm{tr}\left(T_{k|k-1}P_{k|k-1}^{-1}\right)\\ \; & -\frac{1}{2}{\left(x_{k}-{\hat{x}}_{k|k-1}\right)}^{T}P_{k|k-1}^{-1}\left(x_{k}-{\hat{x}}_{k|k-1}\right)+c_{\Xi } \end{array}$$

Using (59) in (52) and letting $\alpha =P_{k|k-1}$, we have:

(60) $$\begin{array}{cc} \log q^{\left(i+1\right)}\left(P_{k|k-1}\right)= & -\frac{1}{2}E^{\left(i\right)}\left[\mathrm{tr}\left(U_{k|k-1}R_{k}^{-1}\right)\right]-\frac{1}{2}E^{\left(i\right)}\left[{\left(z_{k}-h\left(x_{k}\right)\right)}^{T}R_{k}^{-1}\left(z_{k}-h\left(x_{k}\right)\right)\right]\\ \; & -\frac{1}{2}\left({\hat{u}}_{k|k-1}+n_{z}+2\right)E^{\left(i\right)}\left[\log\right|R_{k}\left|\right]-\frac{1}{2}\left({\hat{t}}_{k|k-1}+n_{x}+2\right)\log\left|P_{k|k-1}\right|\\ \; & -\frac{1}{2}\mathrm{tr}\left[\left(A_{k}^{\left(i\right)}+{\hat{T}}_{k|k-1}\right)P_{k|k-1}^{-1}\right]+c_{P_{k|k-1}}\\ = & -\frac{1}{2}\left({\hat{t}}_{k|k-1}+n_{x}+2\right)\log\left|P_{k|k-1}\right|-\frac{1}{2}\mathrm{tr}\left[\left(A_{k}^{\left(i\right)}+{\hat{T}}_{k|k-1}\right)P_{k|k-1}^{-1}\right]+c_{P_{k|k-1}} \end{array}$$

where $q^{\left(i+1\right)}\left(\cdot \right)$ is the approximation of PDF q(·) at the iteration i+1, and $A_{k}^{\left(i\right)}$ is given as follows:

(61) $$\begin{array}{cc} A_{k}^{\left(i\right)}= & E^{\left(i\right)}\left[\left(x_{k}-{\hat{x}}_{k|k-1}\right){\left(x_{k}-{\hat{x}}_{k|k-1}\right)}^{T}\right]\\ = & E^{\left(i\right)}\left[\left(x_{k}-{\hat{x}}_{k|k}^{\left(i\right)}+{\hat{x}}_{k|k}^{\left(i\right)}-{\hat{x}}_{k|k-1}\right){\left(x_{k}-{\hat{x}}_{k|k}^{\left(i\right)}+{\hat{x}}_{k|k}^{\left(i\right)}-{\hat{x}}_{k|k-1}\right)}^{T}\right]\\ = & P_{k|k}^{\left(i\right)}+\left({\hat{x}}_{k|k}^{\left(i\right)}-{\hat{x}}_{k|k-1}\right){\left({\hat{x}}_{k|k}^{\left(i\right)}-{\hat{x}}_{k|k-1}\right)}^{T} \end{array}$$

$q^{\left(i+1\right)}\left(P_{k|k-1}\right)$ is updated as an IW PDF with dof parameter ${\hat{t}}_{k}^{\left(i+1\right)}$ and inverse scale matrix ${\hat{T}}_{k}^{\left(i+1\right)}$:

(62) $$q^{\left(i+1\right)}\left(P_{k|k-1}\right)=\mathrm{IW}\left(P_{k|k-1};{\hat{t}}_{k}^{\left(i+1\right)},{\hat{T}}_{k}^{\left(i+1\right)}\right)$$

where:

(63) $${\hat{t}}_{k}^{\left(i+1\right)}={\hat{t}}_{k|k-1}+1$$

(64) $${\hat{T}}_{k}^{\left(i+1\right)}=A_{k}^{\left(i\right)}+{\hat{T}}_{k|k-1}$$

Let $\alpha =R_{k}$; we have:

(65) $$\log q^{\left(i+1\right)}\left(R_{k}\right)=-0.5\left({\hat{u}}_{k|k-1}+L_{z}+2\right)\log\left|R_{k}\right|-\frac{1}{2}\mathrm{tr}\left[\left(B_{k}^{\left(i\right)}+{\hat{U}}_{k|k-1}\right)R_{k}^{-1}\right]+c_{R_{k}}$$

where $B_{k}^{\left(i\right)}$ is given by:

(66) $$\begin{array}{cc} B_{k}^{\left(i\right)} & =E^{\left(i\right)}\left[\left(z_{k}-h\left(x_{k}\right)\right){\left(z_{k}-h\left(x_{k}\right)\right)}^{T}\right]\\ \; & =\int \left(z_{k}-h\left(x_{k}\right)\right){\left(z_{k}-h\left(x_{k}\right)\right)}^{T}N\left(x_{k};{\hat{x}}_{k|k}^{\left(i\right)},P_{k|k}^{\left(i\right)}\right)dx_{k}\\ \; & =\frac{1}{2n_{x}}\sum_{j=1}^{2n_{x}}\left[\left(z_{k}-h\left(\chi_{k}^{\left(i)(j\right)}\right)\right){\left(z_{k}-h\left(\chi_{k}^{\left(i)(j\right)}\right)\right)}^{T}\right] \end{array}$$

where $\chi_{k}^{\left(i\right)}$ are cubature points based on ${\hat{x}}_{k|k}^{\left(i\right)}$ and $P_{k|k}^{\left(i\right)}$.

(67) $$\left\{\begin{array}{c} \chi_{k}^{\left(i)(j\right)}={\hat{x}}_{k|k}^{\left(i\right)}+\sqrt{n_{x}}{\left(\sqrt{P_{k|k}^{\left(i\right)}}\right)}_{\left(j\right)},\;\left(j=1,2,\cdots ,n_{x}\right)\\ \chi_{k}^{\left(i)(j\right)}={\hat{x}}_{k|k}^{\left(i\right)}-\sqrt{n_{x}}{\left(\sqrt{P_{k|k}^{\left(i\right)}}\right)}_{\left(j-n_{x}\right)},\;\left(j=n_{x}+1,n_{x}+2,\cdots ,2n_{x}\right) \end{array}\right.$$

$q^{\left(i+1\right)}\left(R_{k}\right)$ is updated as an IW PDF with dof parameter ${\hat{u}}_{k}^{\left(i+1\right)}$ and inverse scale matrix ${\hat{U}}_{k}^{\left(i+1\right)}$:

(68) $$q^{\left(i+1\right)}\left(R_{k}\right)=\mathrm{IW}\left(R_{k};{\hat{u}}_{k}^{\left(i+1\right)},{\hat{U}}_{k}^{\left(i+1\right)}\right)$$

where:

(69) $${\hat{u}}_{k}^{\left(i+1\right)}={\hat{u}}_{k|k-1}+1$$

(70) $${\hat{U}}_{k}^{\left(i+1\right)}=B_{k}^{\left(i\right)}+{\hat{U}}_{k|k-1}$$

Let $\alpha =x_{k}$; we have:

(71) $$\begin{array}{cc} \log q^{\left(i+1\right)}\left(x_{k}\right)= & -\frac{1}{2}{\left(x_{k}-{\hat{x}}_{k|k-1}\right)}^{T}E^{\left(i+1\right)}\left[P_{k|k-1}^{-1}\right]\left(x_{k}-{\hat{x}}_{k|k-1}\right)\\ \; & -\frac{1}{2}{\left(z_{k}-h\left(x_{k}\right)\right)}^{T}E^{\left(i+1\right)}\left[R_{k}^{-1}\right]\left(z_{k}-h\left(x_{k}\right)\right)+c_{x} \end{array}$$

where:

(72) $$E^{\left(i+1\right)}\left[R_{k}^{-1}\right]=\left({\hat{u}}_{k}^{\left(i+1\right)}-n_{x}-1\right){\left({\hat{U}}_{k}^{\left(i+1\right)}\right)}^{-1}$$

(73) $$E^{\left(i+1\right)}\left[P_{k|k-1}^{-1}\right]=\left({\hat{t}}_{k}^{\left(i+1\right)}-n_{z}-1\right){\left({\hat{T}}_{k}^{\left(i+1\right)}\right)}^{-1}$$

The one step predicted PDF $p^{\left(i+1\right)}\left(x_{k}|z_{1:k-1}\right)$ and likelihood PDF $p^{\left(i+1\right)}\left(z_{k}|x_{k}\right)$ at iteration i+1 are defined as follows:

(74) $$p^{\left(i+1\right)}\left(x_{k}|z_{1:k-1}\right)=N\left(x_{k};{\hat{x}}_{k|k-1},{\hat{P}}_{k|k-1}^{\left(i+1\right)}\right)$$

(75) $$p^{\left(i+1\right)}\left(z_{k}|x_{k}\right)=N\left(z_{k};h\left(x_{k}\right),{\hat{R}}_{k}^{\left(i+1\right)}\right)$$

where:

(76) $${\hat{P}}_{k|k-1}^{\left(i+1\right)}={\left\{E^{\left(i+1\right)}\left[P_{k|k-1}^{-1}\right]\right\}}^{-1}$$

(77) $${\hat{R}}_{k}^{\left(i+1\right)}={\left\{E^{\left(i+1\right)}\left[R_{k}^{-1}\right]\right\}}^{-1}$$

Employing (74)–(77) in (71), we have:

(78) $$q^{\left(i+1\right)}\left(x_{k}\right)=\frac{1}{c_{k}^{\left(i+1\right)}}p^{\left(i+1\right)}\left(z_{k}|x_{k}\right)p^{\left(i+1\right)}\left(x_{k}|z_{1:k-1}\right)$$

where the normalization constant $c_{k}^{\left(i+1\right)}$ is given as:

(79) $$c_{k}^{\left(i+1\right)}=\int p^{\left(i+1\right)}\left(z_{k}|x_{k}\right)p^{\left(i+1\right)}\left(x_{k}|z_{1:k-1}\right)dx_{k}$$

$q^{\left(i+1\right)}\left(x_{k}\right)$ is updated as the normal distribution with mean ${\hat{x}}_{k|k}^{\left(i+1\right)}$ and variance ${\hat{P}}_{k|k}^{\left(i+1\right)}$:

(80) $$q^{\left(i+1\right)}\left(x_{k}\right)=N\left(x_{k};{\hat{x}}_{k|k}^{\left(i+1\right)},{\hat{P}}_{k|k}^{\left(i+1\right)}\right)$$

where ${\hat{x}}_{k|k}^{\left(i+1\right)}$ and ${\hat{P}}_{k|k}^{\left(i+1\right)}$ at iteration i+1 are calculated similarly to (31)–(37).

The cubature points of $x_{k}$ based on ${\hat{x}}_{k|k-1}$ and modified ${\hat{P}}_{k|k-1}^{\left(i+1\right)}$ are given as:

(81) $$\left\{\begin{array}{c} \chi_{k|k-1}^{\left(i+1)(j\right)}={\hat{x}}_{k|k-1}+\sqrt{n_{x}}{\left(\sqrt{{\hat{P}}_{k|k-1}^{\left(i+1\right)}}\right)}_{\left(j\right)},\;\left(j=1,2,\cdots ,n_{x}\right)\\ \chi_{k|k-1}^{\left(i+1)(j\right)}={\hat{x}}_{k|k-1}-\sqrt{n_{x}}{\left(\sqrt{{\hat{P}}_{k|k-1}^{\left(i+1\right)}}\right)}_{\left(j-n_{x}\right)},\;\left(j=n_{x}+1,n_{x}+2,\cdots ,2n_{x}\right) \end{array}\right.$$

(82) $$Z_{k|k-1}^{\left(i+1)(j\right)}=h\left(\chi_{k|k-1}^{\left(i+1)(j\right)}\right),\left(j=1,2,\cdots ,2n_{x}\right)$$

(83) $${\hat{z}}_{k|k-1}^{\left(i+1\right)}=\frac{1}{2n_{x}}\sum_{j=1}^{2n_{x}}Z_{k|k-1}^{\left(i+1)(j\right)}$$

(84) $$P_{k|k-1}^{zz(i+1)}=\frac{1}{2n_{x}}\sum_{j=1}^{2n_{x}}\left[\left(Z_{k|k-1}^{\left(i+1)(j\right)}-{\hat{z}}_{k|k-1}^{\left(i+1\right)}\right){\left(Z_{k|k-1}^{\left(i+1)(j\right)}-{\hat{z}}_{k|k-1}^{\left(i+1\right)}\right)}^{T}\right]+{\hat{R}}_{k}^{\left(i+1\right)}$$

(85) $$P_{k|k-1}^{xz(i+1)}=\frac{1}{2n_{x}}\sum_{j=1}^{2n_{x}}\left[\left(\chi_{k|k-1}^{\left(i+1)(j\right)}-{\hat{x}}_{k|k-1}\right){\left(Z_{k|k-1}^{\left(i+1)(j\right)}-{\hat{z}}_{k|k-1}^{\left(i+1\right)}\right)}^{T}\right]$$

(86) $$K_{k}^{\left(i+1\right)}=P_{k|k-1}^{xz(i+1)}{\left(P_{k|k-1}^{zz(i+1)}\right)}^{-1}$$

(87) $${\hat{x}}_{k|k}^{\left(i+1\right)}={\hat{x}}_{k|k-1}+K_{k}^{\left(i+1\right)}\left(z_{k}-{\hat{z}}_{k|k-1}^{\left(i+1\right)}\right)$$

(88) $${\hat{P}}_{k|k}^{\left(i+1\right)}=P_{k|k-1}^{\left(i+1\right)}-K_{k}^{\left(i+1\right)}P_{k|k-1}^{zz(i+1)}{\left(K_{k}^{\left(i+1\right)}\right)}^{T}$$

After *N* fixed point iterations, we can obtain the approximate solution of $q(x_{k})$, $q(P_{k|k-1})$ and $q(R_{k})$:

(89) $$q\left(x_{k}\right)\approx q^{\left(N\right)}\left(x_{k}\right)=N\left(x_{k};{\hat{x}}_{k|k}^{\left(N\right)},{\hat{P}}_{k|k}^{\left(N\right)}\right)$$

(90) $$q\left(P_{k|k-1}\right)\approx q^{\left(N\right)}\left(P_{k|k-1}\right)=\mathrm{IW}\left(P_{k|k-1};{\hat{t}}_{k}^{\left(N\right)},{\hat{T}}_{k}^{\left(N\right)}\right)$$

(91) $$q\left(R_{k}\right)\approx q^{\left(N\right)}\left(R_{k}\right)=\mathrm{IW}\left(R_{k};{\hat{u}}_{k}^{\left(N\right)},{\hat{U}}_{k}^{\left(N\right)}\right)$$

When the measurement model is linear, such as the initial alignment measurement model in (12), we can obtain the simplified algorithm, where (66) and (81)–(89) are formulated as follows:

(92) $$\begin{array}{cc} B_{k}^{\left(i\right)}= & E^{\left(i\right)}\left[\left(z_{k}-Hx_{k}\right){\left(z_{k}-Hx_{k}\right)}^{T}\right]\\ = & E^{\left(i\right)}\left[\left(z_{k}-H{\hat{x}}_{k|k}^{\left(i\right)}+H{\hat{x}}_{k|k}^{\left(i\right)}-Hx_{k}\right){\left(z_{k}-H{\hat{x}}_{k|k}^{\left(i\right)}+H{\hat{x}}_{k|k}^{\left(i\right)}-Hx_{k}\right)}^{T}\right]\\ = & \left(z_{k}-H{\hat{x}}_{k|k}^{\left(i\right)}\right){\left(z_{k}-H{\hat{x}}_{k|k}^{\left(i\right)}\right)}^{T}+HP_{k|k}^{\left(i\right)}H^{T} \end{array}$$

(93) $$K_{k}^{\left(i+1\right)}={\hat{P}}_{k|k-1}^{\left(i+1\right)}H^{T}{\left(H{\hat{P}}_{k|k-1}^{\left(i+1\right)}H^{T}+{\hat{R}}_{k}^{\left(i+1\right)}\right)}^{-1}$$

(94) $${\hat{x}}_{k|k}^{\left(i+1\right)}={\hat{x}}_{k|k-1}+K_{k}^{\left(i+1\right)}\left(z_{k}-H{\hat{x}}_{k|k-1}\right)$$

(95) $${\hat{P}}_{k|k}^{\left(i+1\right)}={\hat{P}}_{k|k-1}^{\left(i+1\right)}-K_{k}^{\left(i+1\right)}H{\hat{P}}_{k|k-1}^{\left(i+1\right)}$$

The implementation pseudocode of the proposed adaptive cubature Kalman filter is shown in Algorithm 1.

To implement the proposed ACKF method, we need to select the tuning parameter τ, the forgetting factor ξ, and the iteration number *N*. Tuning parameter τ can be seen as an adjustment parameter of ${\tilde{P}}_{k|k-1}$. If τ is too large, the prior uncertainties induced by nominal ${\tilde{Q}}_{k}$ will influence the measurement update. If τ is too small, the information of the process model will be also lost. According to the research result of [27], the optimal range of the turning parameter is τ∈[2,6], which has better estimation performance and estimation accuracy. The forgetting factor ξ also adjusts the influence of ${\hat{R}}_{k-1}$. Note that ξ=1 means the stationary measurement noise covariance. A large iteration number *N* will improve the estimation accuracy, but also increase the computational cost. According to our experience, N>5 will have good performance in the alignment.
**Algorithm 1**: One-step of the proposed adaptive cubature Kalman filter.**Inputs**: ${\hat{x}}_{k-1|k-1}$, $P_{k-1|k-1}$, $z_{k}$, ${\hat{u}}_{k-1|k-1}$, ${\hat{U}}_{k-1|k-1}$, ${\tilde{Q}}_{k-1}$, τ, ξ, *N*.**Time update**1. Calculate cubature points based on ${\hat{x}}_{k-1|k-1}$ and $P_{k-1|k-1}$.2. $\chi_{k|k-1}^{x(j)}=f\left(\chi_{k-1}^{\left(j\right)}\right),\left(j=1,2,\cdots ,2n_{x}\right)$.3. ${\hat{x}}_{k|k-1}=\frac{1}{2n_{x}}\sum_{j=1}^{2n_{x}}\chi_{k|k-1}^{x(j)}$.4. ${\tilde{P}}_{k|k-1}=\frac{1}{2n_{x}}\sum_{j=1}^{2n_{x}}\left[\left(\chi_{k|k-1}^{x(j)}-{\hat{x}}_{k|k-1}\right){\left(\chi_{k|k-1}^{x(j)}-{\hat{x}}_{k|k-1}\right)}^{T}\right]+g\left({\hat{x}}_{k|k-1}\right){\tilde{Q}}_{k-1}g{\left({\hat{x}}_{k|k-1}\right)}^{T}$.**Iterated measurement update**5. Initialization: ${\hat{x}}_{k|k}^{\left(0\right)}={\hat{x}}_{k|k-1}$, ${\hat{P}}_{k|k}^{\left(0\right)}={\tilde{P}}_{k|k-1}$, ${\hat{T}}_{k|k-1}=\tau {\tilde{P}}_{k|k-1}$,${\hat{t}}_{k|k-1}=n_{x}+\tau +1$,${\hat{u}}_{k|k-1}=\xi \left({\hat{u}}_{k-1|k-1}-n_{z}-1\right)+n_{z}+1$, ${\hat{U}}_{k|k-1}=\xi {\hat{U}}_{k-1|k-1}$.**For**i=0:N−16. Update $q^{\left(i+1\right)}\left(P_{k|k-1}\right)=\mathrm{IW}\left(P_{k|k-1};{\hat{t}}_{k}^{\left(i+1\right)},{\hat{T}}_{k}^{\left(i+1\right)}\right)$,${\hat{t}}_{k}^{\left(i+1\right)}={\hat{t}}_{k|k-1}+1$, ${\hat{T}}_{k}^{\left(i+1\right)}=A_{k}^{\left(i\right)}+{\hat{T}}_{k|k-1}$, where $A_{k}^{\left(i\right)}=P_{k|k}^{\left(i\right)}+\left({\hat{x}}_{k|k}^{\left(i\right)}-{\hat{x}}_{k|k-1}\right){\left({\hat{x}}_{k|k}^{\left(i\right)}-{\hat{x}}_{k|k-1}\right)}^{T}$.7. Update $q^{\left(i+1\right)}\left(R_{k}\right)=\mathrm{IW}\left(R_{k};{\hat{u}}_{k}^{\left(i+1\right)},{\hat{U}}_{k}^{\left(i+1\right)}\right)$,${\hat{u}}_{k}^{\left(i+1\right)}={\hat{u}}_{k|k-1}+1$, ${\hat{U}}_{k}^{\left(i+1\right)}=B_{k}^{\left(i\right)}+{\hat{U}}_{k|k-1}$, where $B_{k}^{\left(i\right)}=\left(z_{k}-H{\hat{x}}_{k|k}^{\left(i\right)}\right){\left(z_{k}-H{\hat{x}}_{k|k}^{\left(i\right)}\right)}^{T}+HP_{k|k}^{\left(i\right)}H^{T}$.8. Update $q^{\left(i+1\right)}\left(x_{k}\right)=N\left(x_{k};{\hat{x}}_{k|k}^{\left(i+1\right)},{\hat{P}}_{k|k}^{\left(i+1\right)}\right)$,${\hat{P}}_{k|k-1}^{\left(i+1\right)}={\left(\left({\hat{t}}_{k}^{\left(i+1\right)}-n_{x}-1\right){\left({\hat{T}}_{k}^{\left(i+1\right)}\right)}^{-1}\right)}^{-1}$, ${\hat{R}}_{k}^{\left(i+1\right)}={\left(\left({\hat{u}}_{k}^{\left(i+1\right)}-n_{z}-1\right){\left({\hat{U}}_{k}^{\left(i+1\right)}\right)}^{-1}\right)}^{-1}$.9. Calculate the mean and variance of posterior PDF,$K_{k}^{\left(i+1\right)}={\hat{P}}_{k|k-1}^{\left(i+1\right)}H^{T}{\left(H{\hat{P}}_{k|k-1}^{\left(i+1\right)}H^{T}+{\hat{R}}_{k}^{\left(i+1\right)}\right)}^{-1}$,${\hat{x}}_{k|k}^{\left(i+1\right)}={\hat{x}}_{k|k-1}+K_{k}^{\left(i+1\right)}\left(z_{k}-H{\hat{x}}_{k|k-1}\right)$,${\hat{P}}_{k|k}^{\left(i+1\right)}={\hat{P}}_{k|k-1}^{\left(i+1\right)}-K_{k}^{\left(i+1\right)}H{\hat{P}}_{k|k-1}^{\left(i+1\right)}$.**End for**10. ${\hat{x}}_{k|k}={\hat{x}}_{k|k}^{\left(N\right)}$,$P_{k|k}={\hat{P}}_{k|k}^{\left(N\right)}$, ${\hat{u}}_{k|k}={\hat{u}}_{k}^{\left(N\right)}$, ${\hat{U}}_{k|k}={\hat{U}}_{k}^{\left(N\right)}$.**Outputs**: ${\hat{x}}_{k|k}$, $P_{k|k}$, ${\hat{u}}_{k|k}$, ${\hat{U}}_{k|k}$.

## 4. Simulation and Vehicle Experiment of SINS

### 4.1. Simulation

**Figure 2:**
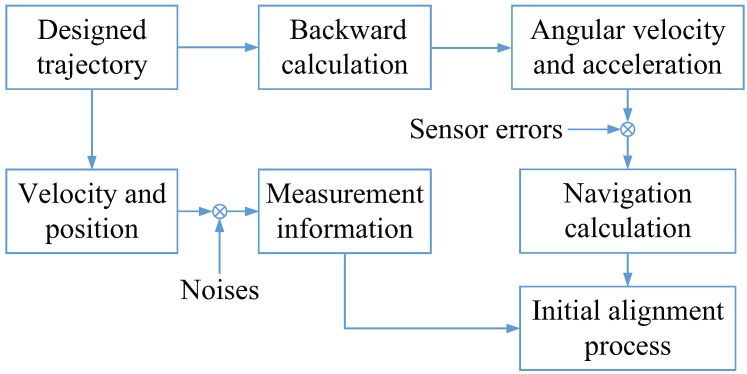
The block diagram of the simulation process.

Firstly, the simulation is given as follows. Through the designed trajectory of the carrier, the outputs of the gyroscope and accelerometer with errors could be obtained according to their mathematic models. In addition, by adding errors into the true velocity and position of the trajectory, the measurements for initial alignment were built as the output of a virtual GPS receiver. The simulation process can be summarized as the following block diagram which is shown in Figure 2.

The initial latitude and longitude of the carrier were set as 45.776 ${}^{\circ }N$ and 126.446 ${}^{\circ }E$, respectively. The constant velocity was set as 10 m/s. The motion of the carrier was set as the typical swing process based on the sine function:

(96) $$\left\{\begin{array}{c} pitch=pitch_{m}\sin\left(2\pi kT_{s}/T_{p}+pitch_{0}\right)+pitchI\\ roll=roll_{m}\sin\left(2\pi kT_{s}/T_{R}+roll_{0}\right)+rollI\\ heading=heading_{m}\sin\left(2\pi kT_{s}/T_{H}+heading_{0}\right)+headingI \end{array}\right\}$$

where $pitch_{m}$, $roll_{m}$, and $heading_{m}$ are swing amplitudes, which were selected as $pitch_{m}=5^{\circ }$, $roll_{m}=6^{\circ }$, and $heading_{m}=7^{\circ }$; $T_{p}$, $T_{R}$, and $T_{H}$ are swing periods, which were selected as $T_{p}=7$ s, $T_{R}=8$ s, and $T_{H}=9$ s; $pitch_{0}$, $roll_{0}$, and $heading_{0}$ are initial swing phases, which were selected as $pitch_{0}=0^{\circ }$, $roll_{0}=0^{\circ }$, and $heading_{0}=0^{\circ }$; pitchI, rollI, and headingI are initial attitude angles, which were selected as $pitchI=0^{\circ }$, $rollI=0^{\circ }$, and $headingI=45^{\circ }$; $T_{s}=0.01$ s is the discrete time of the system. The initial misalignment angles were set as $\phi =[5^{\circ };5^{\circ };15^{\circ }]$. The nominal sensor specifications of SINS in the simulation were set as the practical SINS, which are shown in Table 1.

Based on the parameters in Table 1, the nominal state noise covariance matrix was set as ${\tilde{Q}}_{k}=T_{s}\mathrm{diag}{\left(\left[0.1^{\circ }/h\left(\sqrt{s}\right)I_{3\times 3};10^{-5}g\left(\sqrt{s}\right)I_{2\times 2};0_{7\times 1}\right]\right)}^{2}$. The nominal measurement noise covariance matrix was set as ${\tilde{R}}_{k}=\mathrm{diag}{\left(\left[0.1m/sI_{2\times 2};\arctan\left(10m/R_{e}\right)I_{2\times 2}\right]\right)}^{2}$. Considering that the true $Q_{k}$ was always larger than the nominal value due to the external disturbance such as vibration and the slowly time varying characteristic, the true $Q_{k}$ was set as $Q_{k}=\left[1+0.1\cos\left(\frac{\pi kT_{s}}{H_{n}}\right)\right]T_{s}\mathrm{diag}{\left(\left[1^{\circ }/h\left(\sqrt{s}\right)I_{3\times 3};10^{-4}g\left(\sqrt{s}\right)I_{2\times 2};0_{7\times 1}\right]\right)}^{2}$, where $H_{n}=100s$ denotes the simulation time. The true $R_{k}$ was set as $R_{k}=\left[1+0.1\cos\left(\frac{\pi kT_{s}}{H_{n}}\right)\right]\mathrm{diag}$${\left(\left[0.01m/sI_{2\times 2};\arctan\left(1m/R_{e}\right)I_{2\times 2}\right]\right)}^{2}$. Existing UKF [18], CKF [14], the Sage–Husa adaptive Kalman filter (SHKF) [26], and CKF with true noise covariance matrices (TCKF) were selected to compare the performance with the proposed ACKF method. For the proposed ACKF, the tuning parameter, forgetting factor, and iteration number were set as τ=5, ξ=0.98, and N=10, respectively. The initial state was set as ${\hat{x}}_{0|0}=0_{12\times 1}$. The initial state error covariance matrix was set as $P_{0|0}=\mathrm{diag}{\left(\left[5^{\circ };5^{\circ };15^{\circ };0.1m/sI_{2\times 2};\arctan\left(10m/R_{e}\right)I_{2\times 2};0.01^{\circ }/hI_{3\times 3};10^{-4}gI_{2\times 2}\right]\right)}^{2}$. The frequency of measurement updating was set as 10 Hz. The total number of Monte Carlo runs was set as M=30. Furthermore, to evaluate the estimation accuracy of $P_{k|k-1}$ and $R_{k}$, the square root of normalized Frobenius norm (SRNFN) was used, which is defined as follows:

(97) $$\left\{\begin{array}{c} SRNFN_{P}={\left(\frac{1}{n_{x}^{2}M}\sum_{m=1}^{M}{∥{\hat{P}}_{k|k-1}^{m}-{\bar{P}}_{k|k-1}^{m}∥}^{2}\right)}^{\frac{1}{4}}\\ SRNFN_{R}={\left(\frac{1}{n_{z}^{2}M}\sum_{m=1}^{M}{∥{\hat{R}}_{k}^{m}-R_{k}^{m}∥}^{2}\right)}^{\frac{1}{4}} \end{array}\right.$$

where ${\bar{P}}_{k|k-1}$ means the accurate one step predicted state error covariance matrix provided by TCKF.

**Figure 3:**
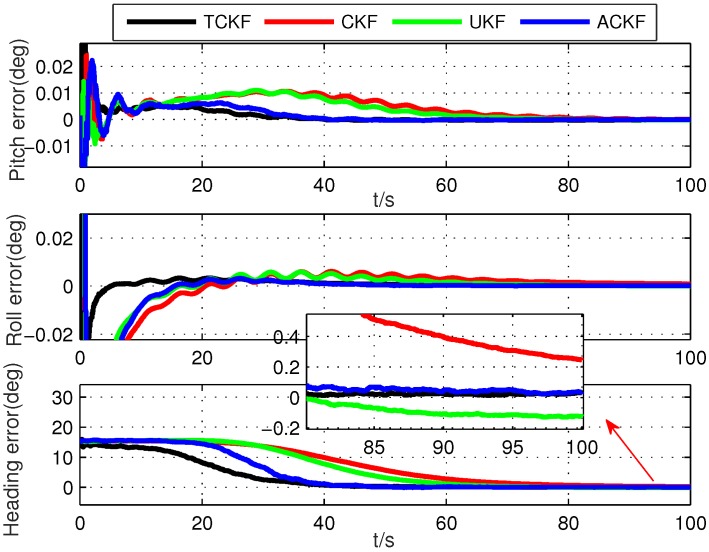
Alignment errors of different methods. TCKF, Kalman filter with true noise covariance matrices; ACKF, adaptive cubature Kalman filter.

**Figure 4:**
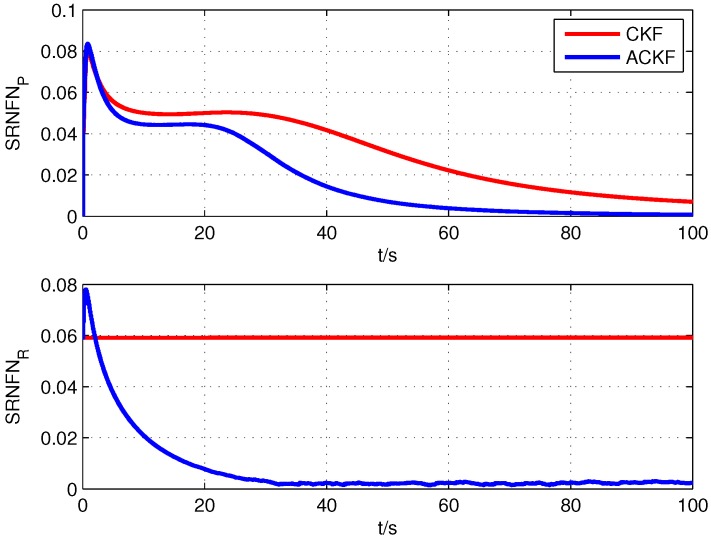
Square root of normalized Frobenius norms (SRNFNs) of $P_{k|k-1}$ and $R_{k}$.

The alignment errors of different methods are shown in Figure 3. The average alignment errors in the latter 20 s are shown in Table 2. The results of SRNFNs are shown in Figure 4. Note that due to the bad stability of SHKF, its simulation results are not shown in the following simulation. It was because that when the error covariance matrices of the state model and measurement model needed to be estimated simultaneously, the estimation accuracy of SHKF was poor, and its stability was heavily influenced by the non-positive definite of noise covariance matrices.

From the results of simulation, it can be seen from Figure 3 that the proposed ACKF had better alignment accuracy than the existing CKF and UKF, and its alignment error was close to the error from TCKF. This was because the proposed ACKF could adaptively estimate $P_{k|k-1}$ and $R_{k}$ and eliminate the influence of the inaccurate noise covariance information. It also can be seen from Figure 4 that the proposed ACKF had smaller SRNFNs than the existing CKF and UKF. In the conventional Kalman filter, $P_{k|k-1}$ represents the predicted error based on the measurement information $z_{1:k-1}$. Due to the inaccurate noise covariance matrices of the state and measurement, the conventional filters relied on the wrong information. Thus, the convergence speed and estimation accuracy of CKF and UKF were lower than those of TCKF and ACKF.

To further discuss the influence of the parameters used in the proposed ACKF, simulations with different tuning parameters τ, forgetting factors ξ, and iteration numbers *N* were conducted.

Table 3, Table 4 and Table 5 show respectively the estimation errors with different τ, ξ, and *N*. It can be seen that the proposed ACKF had a consistent estimation performance when τ∈[2,6], ξ=0.96,0.97,0.98,0.99 and N>5, which also corresponded to the aforementioned analyses.

### 4.2. Vehicle Experiment

**Figure 5:**
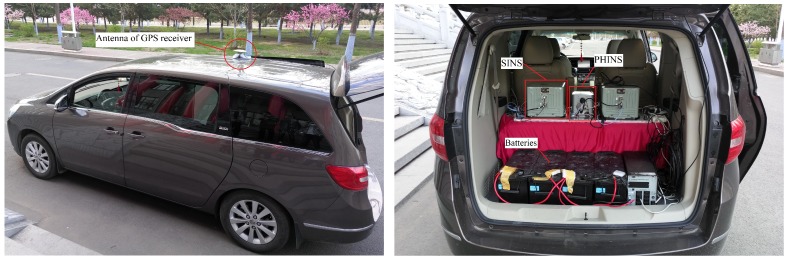
The experimental setup of the vehicle experiment.

Secondly, a vehicle experiment was performed to validate the performance of the proposed ACKF method in practice. The photonics inertial navigation system (PHINS) produced by the company IXSEA France, a self-made SINS, and the antenna of GPS receiver were mounted on the car as shown in Figure 5.

**Figure 6:**
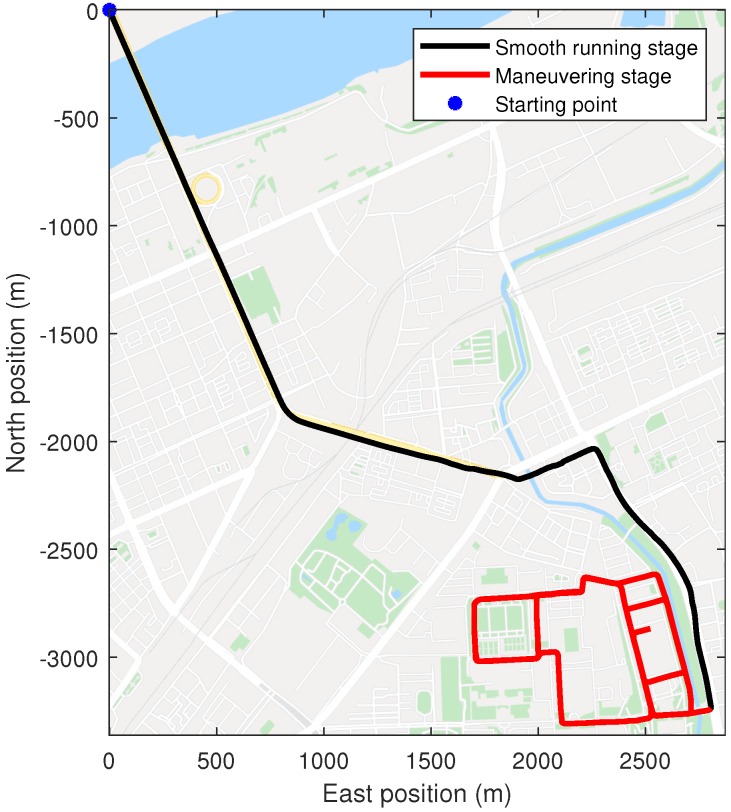
Vehicle experiment trajectory.

**Figure 7:**
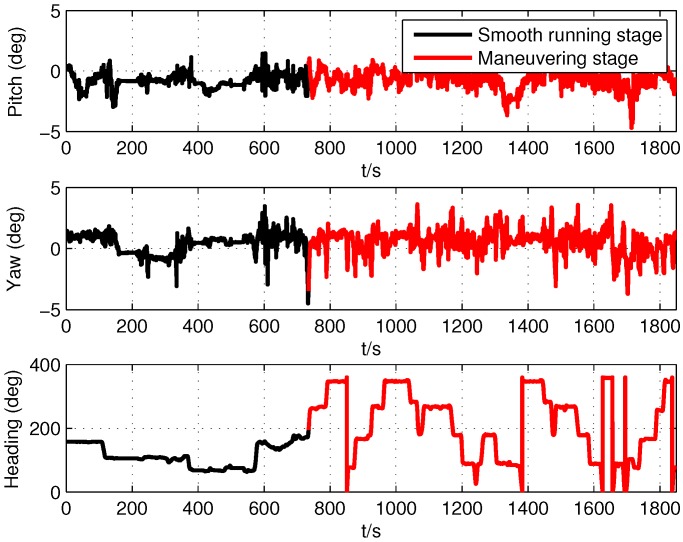
Vehicle experiment attitude.

**Figure 8:**
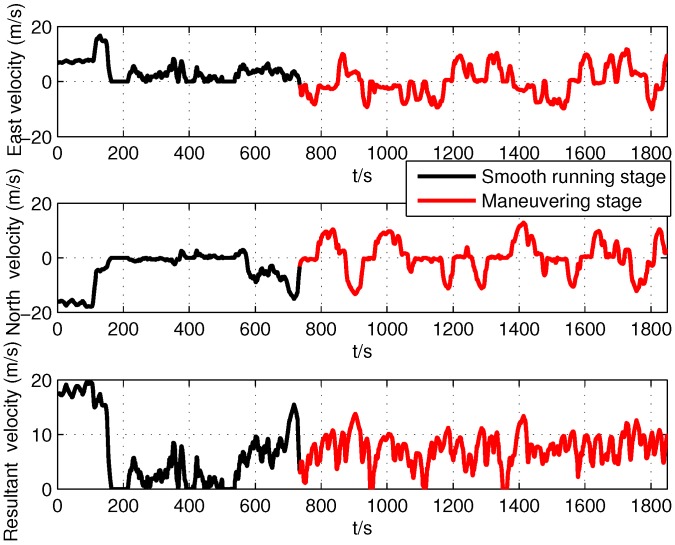
Vehicle experiment velocity.

The self-made SINS had a three axis fiber optic gyroscope and accelerometer to measure body angular rate and specific force, respectively. The theoretical sensor specifications of SINS were the same as the simulation in Table 1. The GPS receiver could provide position and Doppler derived velocity measurements to carry out initial alignment and integrate with PHINS to constitute a high accuracy attitude reference system for SINS. The lever arm and the installation error angles between SINS and PHINS were compensated. The output frequency of SINS and PHINS/GPS integrated navigation system were 100 Hz and 10 Hz, respectively. The root mean square of the attitude accuracy of the PHINS/GPS integrated navigation system was $0.01^{\circ }$ for roll and pitch angles and $0.01^{\circ }\sec\left(L\right)$ for the heading angle. Because of the uncertainty of the sensor parameters of SINS and the influence of external disturbance in motion conditions, we could not obtain the accurate sensor parameters, which determined the state noise covariance values in the Kalman filter. Therefore, the theoretical sensor parameters were nominal and inaccurate in the practical environment. The experiment was carried out in an urban area (45.776${}^{\circ }\;N$, 126.446${}^{\circ }\;E$), and the running stage continued for 1850 s, including two parts: smooth running stage (0 s to 735 s) and maneuvering stage (735 s to 1850 s). The running trajectory, attitude, and velocity of the car provided by the PHINS/GPS integrated navigation system are shown in Figure 6, Figure 7 and Figure 8, respectively. It can be seen that the car had frequent turn movements in the severe maneuvering stage, which would subsequently induce the uncertain sensor parameters of SINS. Therefore, the data of this running stage could better verify the effectiveness of the proposed ACKF method.

**Figure 9:**
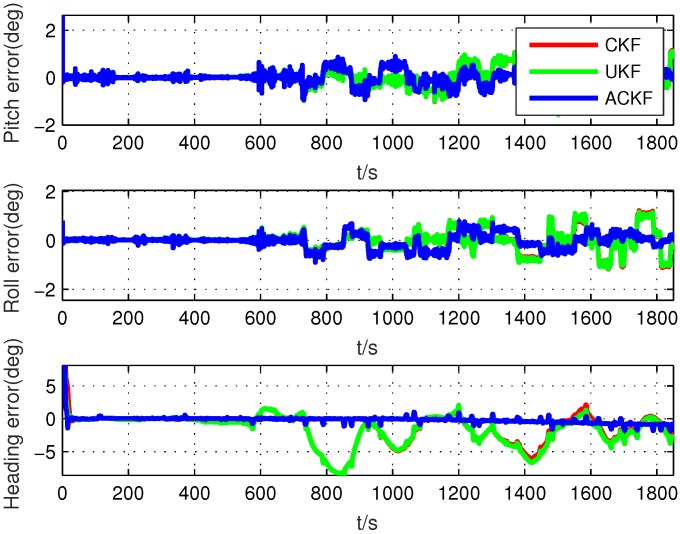
Alignment errors in the whole test.

Normally, the initial attitude matrix of SINS was set as the identity matrix. However, this may not induce the large misalignment angles sometimes. Therefore, the additional large misalignment angles, which were set as $\phi =[5^{\circ };5^{\circ };15^{\circ }]$, were added into the reference initial attitude matrix of PHINS/GPS integrated navigation system to make up the initial attitude matrix of SINS. The nominal state noise covariance matrix ${\tilde{Q}}_{k}$ and nominal measurement noise covariance matrix ${\tilde{R}}_{k}$ were set the same as the simulation. The tuning parameter, forgetting factor, and iteration number were set as τ=5, ξ=0.98, and N=10, respectively. Firstly, to compare the performance of the proposed ACKF and the existing methods, we used the whole data to simulate the initial alignment process. Because the true noise covariance matrices were unknown in the practical environment, the calibration results of SINS as shown in Table 1 were selected as the parameters of the filters. The alignment errors of these methods are shown in Figure 9. From the results of the experiment, it can be seen that the performances of ACKF, UKF, and CKF were similar in the smooth running stage, which was because the preset nominal values were close to the true values for the high accuracy SINS. However, in the maneuvering stage, the performances of UKF and CKF were worse than the proposed ACKF, especially the heading angle error, which is because the noise covariance matrices were changing when the carrier was maneuvering, which had a severe influence on the alignment accuracy.

**Figure 10:**
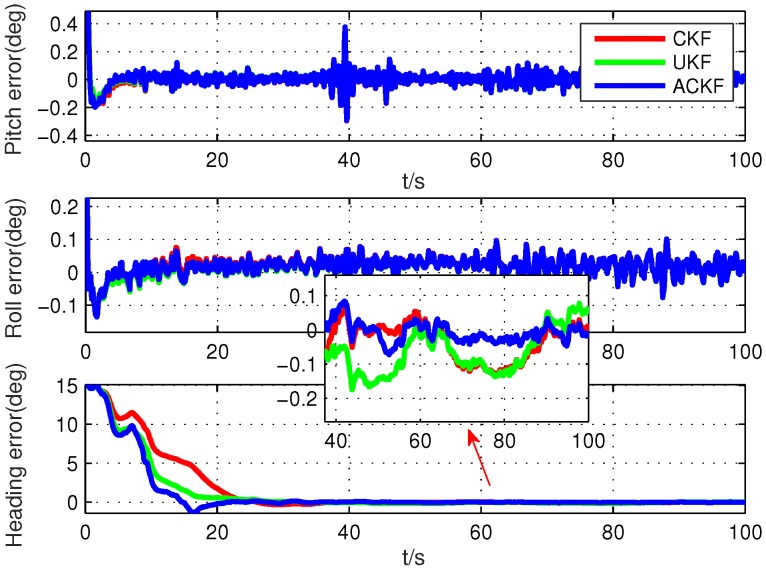
Alignment errors in the smooth segment of 0 s to 100 s.

**Figure 11:**
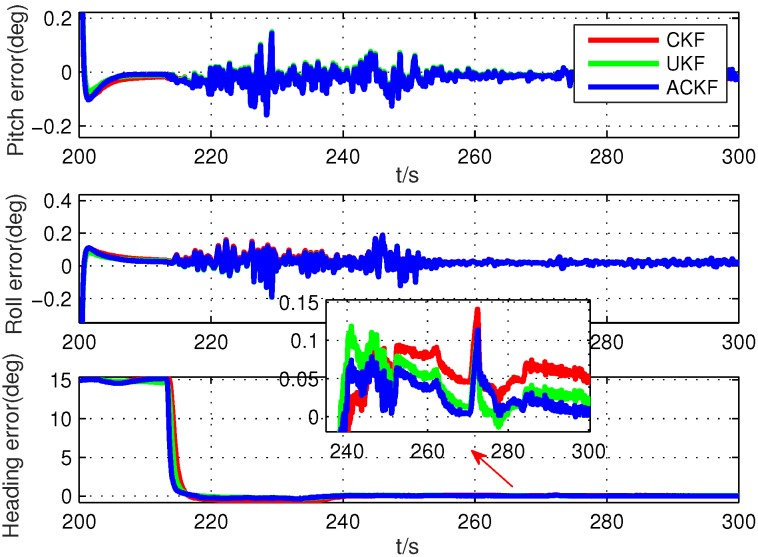
Alignment errors in the smooth segment of 200 s to 300 s.

**Figure 12:**
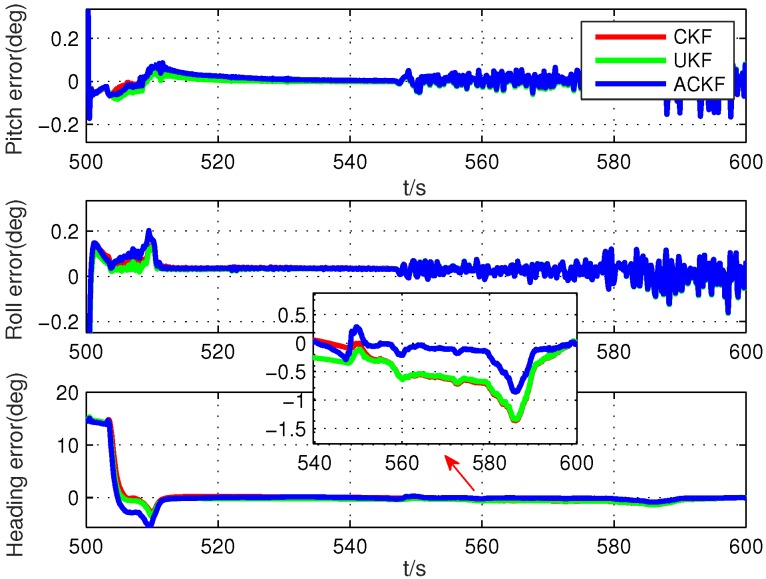
Alignment errors in the smooth segment of 500 s to 600 s.

**Figure 13:**
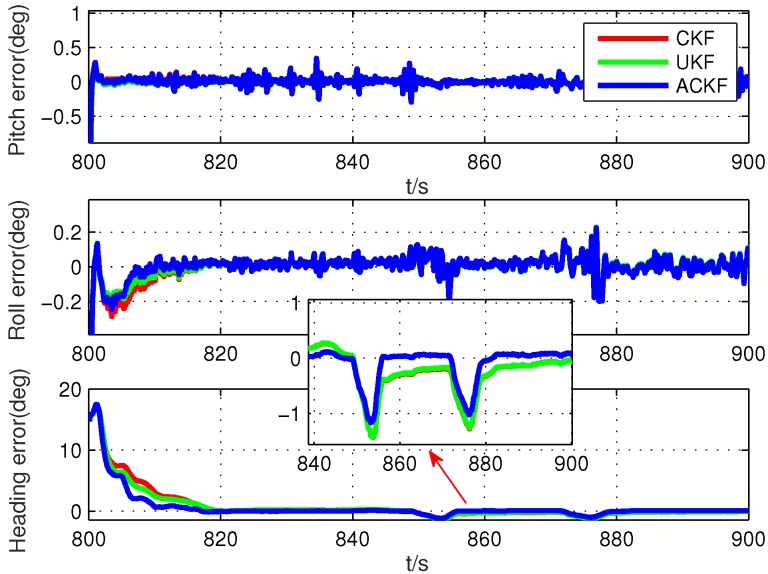
Alignment errors in the maneuvering segment of 800 s to 900 s.

**Figure 14:**
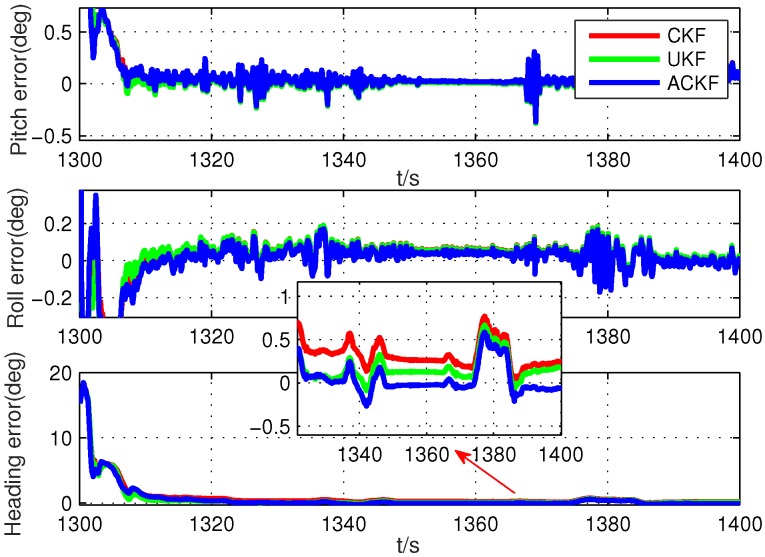
Alignment errors in the maneuvering segment of 1300 s to 1400 s.

**Figure 15:**
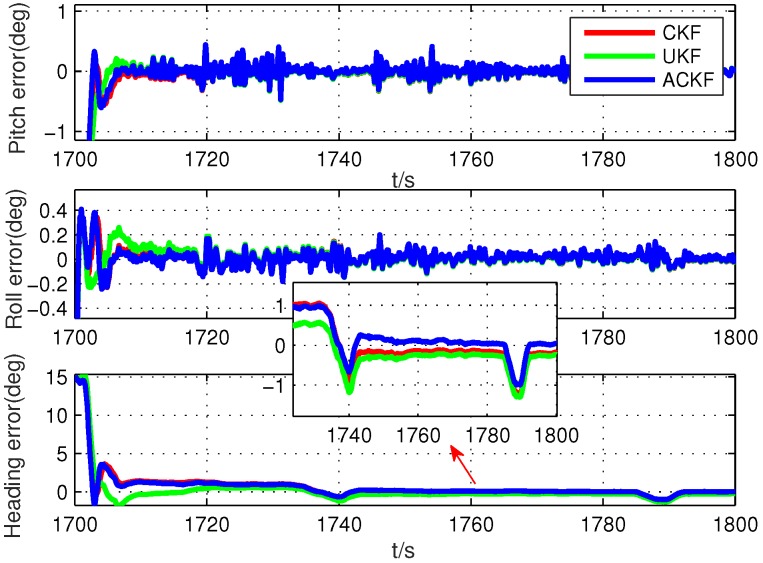
Alignment errors in the maneuvering segment of 1700 s to 1800 s.

Secondly, because the alignment times were very short, we selected six different segments in the whole test data, including smooth segments (0 s to 100 s, 200 s to 300 s, 500 s to 600 s) and maneuvering segments (800 s to 900 s, 1300 s to 1400 s, 1700 s to 1800 s). The alignment errors of these methods are shown in Figure 10, Figure 11, Figure 12, Figure 13, Figure 14 and Figure 15, where Figure 10, Figure 11 and Figure 12 are the results in the smooth segment and Figure 13, Figure 14 and Figure 15 are the results in the maneuvering segment. The average alignment errors in the latter 20 s are shown in Table 6 and Table 7.

According to Figure 10, Figure 11, Figure 12, Figure 13, Figure 14 and Figure 15 and Table 6 and Table 7, it can be seen that the proposed ACKF always had better performance than UKF and CKF. This was because the proposed ACKF could better estimate the measurement noise covariance matrix and the one step prediction covariance matrix influenced by the state noise covariance matrix. Besides, combined with the heading angle curve in Figure 7, we can find that, when the car was making a turn, the performance of these three methods was becoming worse simultaneously. That was because the residual scale error and dynamic error of initial sensors in the turning process were large and would degrade the accuracy of initial alignment. However, the proposed ACKF could quickly converge when the turning process ended. Therefore, compared with the traditional methods and existing adaptive methods, we could conclude that the proposed ACKF had better stability and estimation accuracy, which could eliminate the influence of the uncertain state noise covariance matrix and measurement noise covariance matrix.

## 5. Conclusions

This paper proposed a novel adaptive cubature Kalman filter based variational Bayesian method to solve the alignment problem of SINS with initial large misalignment angles and uncertain noise covariance matrices. The one step predicted error covariance matrix and measurement noise covariance matrix were adaptively estimated together. Simulation and vehicle experiment results illustrated that the proposed ACKF had better stability and estimation accuracy than the existing adaptive filter methods and traditional nonlinear filter methods.

## Figures and Tables

**Table 1:** The nominal sensor specifications of the strapdown inertial navigation system (SINS).

Parameters	Value
Gyroscope bias stability	$0.01^{\circ }/$h
Gyroscope angular random walk	$0.1^{\circ }$/h($\sqrt{s})$
Accelerometer bias stability	$10^{-4}g$
Accelerometer velocity random walk	$10^{-5}g\left(\sqrt{s}\right)$

**Table 2:** Average alignment errors of different methods in the latter 20 s.

Methods	Pitch	Roll	Heading
TCKF	$0.0001^{\circ }$	$0.0001^{\circ }$	$0.0214^{\circ }$
UKF	$-0.0003^{\circ }$	$-0.0003^{\circ }$	$-0.1177^{\circ }$
CKF	$0.0002^{\circ }$	$0.0010^{\circ }$	$0.3119^{\circ }$
ACKF	$0.0001^{\circ }$	$0.0002^{\circ }$	$0.0283^{\circ }$

**Table 3:** Average alignment errors with different tuning parameters τ (ξ=0.98 and N=10).

Parameters	Value	Pitch	Roll	Heading
τ	τ=2	$0.0001^{\circ }$	$0.0001^{\circ }$	$0.0267^{\circ }$
	τ=3	$0.0001^{\circ }$	$0.0002^{\circ }$	$0.0275^{\circ }$
	τ=4	$0.0001^{\circ }$	$0.0002^{\circ }$	$0.0279^{\circ }$
	τ=5	$0.0001^{\circ }$	$0.0002^{\circ }$	$0.0289^{\circ }$
	τ=6	$0.0002^{\circ }$	$0.0003^{\circ }$	$0.0302^{\circ }$

**Table 4:** Average alignment errors with different forgetting factors ξ (τ=5 and N=10).

Parameters	Value	Pitch	Roll	Heading
ξ	ξ=0.96	$0.0001^{\circ }$	$0.0001^{\circ }$	$0.0262^{\circ }$
	ξ=0.97	$0.0001^{\circ }$	$0.0001^{\circ }$	$0.0266^{\circ }$
	ξ=0.98	$0.0001^{\circ }$	$0.0002^{\circ }$	$0.0284^{\circ }$
	ξ=0.99	$0.0002^{\circ }$	$0.0002^{\circ }$	$0.0289^{\circ }$

**Table 5:** Average alignment errors with different iteration numbers *N* (τ=5 and ξ=0.98).

Parameters	Value	Pitch	Roll	Heading
*N*	N=5	$0.0002^{\circ }$	$0.0003^{\circ }$	$0.0463^{\circ }$
	N=10	$0.0001^{\circ }$	$0.0001^{\circ }$	$0.0286^{\circ }$
	N=20	$0.0001^{\circ }$	$0.0001^{\circ }$	$0.0278^{\circ }$
	N=30	$0.0001^{\circ }$	$0.0001^{\circ }$	$0.0263^{\circ }$

**Table 6:** Average alignment errors in the smooth segment.

Segments	Methods	Pitch	Roll	Heading
0 s to 100 s	UKF	$0.005^{\circ }$	$0.016^{\circ }$	$-0.015^{\circ }$
	CKF	$0.004^{\circ }$	$0.016^{\circ }$	$-0.040^{\circ }$
	ACKF	$0.006^{\circ }$	$0.015^{\circ }$	$-0.013^{\circ }$
200 s to 300 s	UKF	$-0.014^{\circ }$	$0.022^{\circ }$	$0.026^{\circ }$
	CKF	$-0.014^{\circ }$	$0.021^{\circ }$	$0.058^{\circ }$
	ACKF	$-0.013^{\circ }$	$0.020^{\circ }$	$0.014^{\circ }$
500 s to 600 s	UKF	$-0.010^{\circ }$	$0.005^{\circ }$	$-0.594^{\circ }$
	CKF	$-0.010^{\circ }$	$0.005^{\circ }$	$-0.607^{\circ }$
	ACKF	$-0.009^{\circ }$	$0.008^{\circ }$	$-0.303^{\circ }$

**Table 7:** Average alignment errors in the maneuvering segment.

Segments	Methods	Pitch	Roll	Heading
800 s to 900 s	UKF	$-0.002^{\circ }$	$0.006^{\circ }$	$-0.127^{\circ }$
	CKF	$-0.003^{\circ }$	$0.006^{\circ }$	$-0.126^{\circ }$
	ACKF	$-0.002^{\circ }$	$0.001^{\circ }$	$0.060^{\circ }$
1300 s to 1400 s	UKF	$0.081^{\circ }$	$0.011^{\circ }$	$0.186^{\circ }$
	CKF	$0.083^{\circ }$	$0.011^{\circ }$	$0.265^{\circ }$
	ACKF	$0.075^{\circ }$	$-0.004^{\circ }$	$0.028^{\circ }$
1700 s to 1800 s	UKF	$-0.013^{\circ }$	$0.006^{\circ }$	$-0.489^{\circ }$
	CKF	$-0.014^{\circ }$	$0.005^{\circ }$	$-0.415^{\circ }$
	ACKF	$-0.012^{\circ }$	$0.011^{\circ }$	$-0.186^{\circ }$
